# Comparative Effects of Red and Blue LED Light on Melatonin Levels During Three-Hour Exposure in Healthy Adults

**DOI:** 10.3390/life15050715

**Published:** 2025-04-28

**Authors:** Ana Sanchez-Cano, María José Luesma-Bartolomé, Estela Solanas, Elvira Orduna-Hospital

**Affiliations:** 1 Department of Applied Physics, University of Zaragoza, 50009 Zaragoza, Spain; 2 Aragon Institute for Health Research (IIS Aragon), 50009 Zaragoza, Spain; 3 Department of Human Anatomy and Histology, University of Zaragoza, 50009 Zaragoza, Spain; mjluesma@unizar.es (M.J.L.-B.); esolanas@unizar.es (E.S.)

**Keywords:** circadian rhythms, melatonin, integrative lighting, LED light, blue light, red light, non-visual effects, salivary biomarkers

## Abstract

Circadian rhythms, essential for regulating human physiology and behavior, are influenced by light exposure, particularly at night. This study examined the impact of red (631 nm) and blue (464 nm) LED light on melatonin secretion, a key circadian marker. Twelve participants aged 19–55 years were exposed to red and blue light for three hours (9:00 p.m.–midnight), with hourly saliva samples analyzed via ELISA to track melatonin levels. Initially, melatonin levels were comparable under both light conditions. After one hour, both lights suppressed melatonin, but differences emerged after two hours: blue light-maintained suppression, with levels at 7.5 pg/mL, while red light allowed recovery to 26.0 pg/mL (*p* = 0.019). This pattern persisted at the third hour. Blue light had stronger suppression effects, particularly in younger participants and men. These results underscore blue light’s disruptive effects on circadian health and highlight red light as a less disruptive alternative for nighttime environments.

## 1. Introduction

Human physiology and behavior are governed by circadian rhythms, which are synchronized to the 24-h solar day by the circadian clock located in the suprachiasmatic nucleus (SCN) of the hypothalamus. The SCN receives environmental light information via the retinohypothalamic tract (RHT), aligning internal biological time with the external light-dark cycle. This synchronization underlies various physiological processes, such as hormone secretion, body temperature regulation, and cognitive performance, even in the absence of external cues, melatonin production, alertness, and cognitive functions, collectively referred to as non-visual effects of light [1,2,3]. These effects are mediated by a photoreceptive system that includes rods, cones, and the recently known intrinsically photosensitive retinal ganglion cells (ipRGCs), which express the photopigment melanopsin [4,5]. Advancements in physiology have transformed our understanding of retinal structure and its neural connections. While the classical visual photoreceptors, rods and cones, have been well-studied, the ipRGCs have been uncovered [4,6]. Their photosensitivity is driven by the photopigment melanopsin, which exhibits peak sensitivity in the short-wavelength region of the visible spectrum and encodes ambient light intensity independent of rods and cones [7,8]. The characteristics and responses of all five photoreceptor types have been extensively documented [9]. The ipRGCs established their role in light-induced melatonin suppression during the biological night, emphasizing their importance in non-image-forming (NIF) processes such as circadian rhythm regulation, the pupillary light reflex (PLR), and the modulation of sleep, mood, and alertness [10,11,12]. Emerging evidence suggests interconnected roles among photoreceptors, rather than strictly independent functions [13]. Disruptions in light exposure, particularly at night, can desynchronize circadian rhythms, as seen in shift work, jet lag, and prolonged evening light exposure, delaying sleep onset and suppressing sleepiness. Optimizing light exposure is essential to enhance health benefits while mitigating adverse effects on circadian health [14].

These effects vary significantly between individuals, influenced by factors such as timing, intensity, wavelength, and prior light exposure history [15,16,17,18,19]. These individual differences in the NIF effects of light via ipRGCs are shaped by age, health, sensitivity, and light characteristics. Younger adults respond to dim, colored light with heart rate changes, whereas older adults require higher intensities, indicating age-related sensitivity decline [20]. This also has clinical implications; desynchronized circadian rhythms in the elderly often reduce light therapy efficacy, highlighting the need to study ipRGC function [11]. This research is crucial for developing targeted light therapies to improve cognitive function, mood, and quality of life, especially as conditions such as diabetic retinopathy impair ipRGC function, disrupting circadian rhythms and sleep [11,21].

The CIE S 026/E:2018 international standard by the International Commission on Illumination (CIE) marked a key advancement, defining spectral weighting functions for cones, rods, and melanopsin to standardize light measurement for NIF effects [22,23]. This milestone supports mechanistic models of circadian and neuroendocrine phototransduction and has enabled the development of consensus guidelines for optimizing light exposure to enhance sleep, alertness, and overall health [14]. These recommendations are derived from a meta-analysis of data from healthy young adults [24]. The guidelines propose a minimum melanopic Equivalent Daylight Illuminance (mEDI) of 250 melanopic lux at the eye during daytime hours, with a maximum of 10 mEDI during the three hours preceding bedtime. During sleep, it should not exceed 1 mEDI; however, if visual tasks are required during nighttime hours, the melanopic mEDI can be increased to a maximum of 10 lux [14,23].

Meanwhile, recommendation by the WELL building standard [25] ensures occupants receive adequate light exposure, in Equivalent Melanopic Light (EML), to support circadian health by aligning rhythms with the natural day-night cycle, addressing deficiencies caused by indoor environments with insufficient lighting. For places during the daytime, electric lighting should be designed to achieve thresholds of at least 150 EML (136 mEDI) or 120 EML (109 mEDI) for the minimum standard for compliance, and at least 275 EML (250 mEDI) or 180 EML (163 mEDI) for a more advanced level of performance, measured on the vertical plane at eye level for at least four hours by noon, ensuring effective stimulation of the circadian system while avoiding overstimulation at night.

On the other hand, the revised model proposed by Rea et al. [26,27] has been widely used in research papers since it optimizes the CL_A_ (Circadian Light) spectral sensitivity framework while preserving key features: the relationship between (Circadian Stimuli) CS and predicted nocturnal melatonin suppression, such that a CS of 0.5 corresponds to 50% melatonin suppression after a 1-h reference light exposure [28].

This preliminary study aims to compare the effects of two LED light exposures on nighttime melatonin levels in human saliva, utilizing antibody-based assays such as ELISA, which are considered the gold standard for measuring salivary biomarkers [2]. These two selected LEDs were fully characterized using the three models previously described, providing a robust basis for evaluating their impact on circadian regulation. By measuring melatonin concentration changes under each light condition, we seek to clarify how different spectra could influence melatonin regulation and circadian rhythms, with implications for optimizing lighting environments for health and well-being.

## 2. Materials and Methods

### 2.1. Experimental Setup Configuration

**Figure 1:**
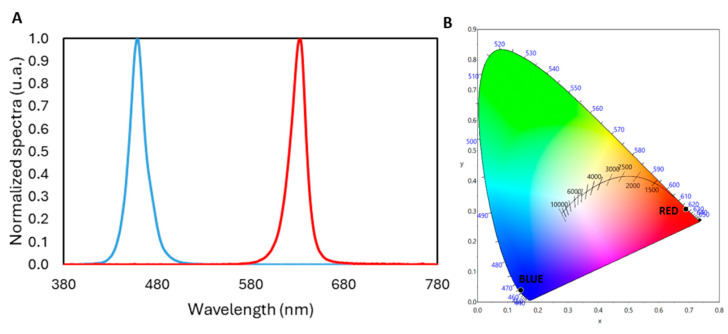
Spectral power distribution (**A**) and CIE 1931 chromaticity diagram (**B**) for the two LED light sources used in this study. (**A**) The blue LED shows a peak in the short-wavelength region (464 nm), while the red LED peaks in the long-wavelength region (631 nm). (**B**) Chromaticity coordinates plotted on the CIE diagram indicate the distinct color characteristics of each LED, corresponding to their spectral peaks.

To determine the spectral power distribution (SPD) and total irradiance (W·m^−2^) of the two selected LEDs used in the experiments, a calibrated spectroradiometer (model StellarNet-Black Comet, StellarNet, Inc., Tampa, FL, USA) was used. A blue LED with a peak emission wavelength of 464 nm and a full width at half maximum (FWHM) of 24 nm, and a red LED with a peak emission wavelength of 631 nm and an FWHM of 18 nm were selected (see Figure 1). Other characteristics are specified in Table 1.

**Figure 2:**
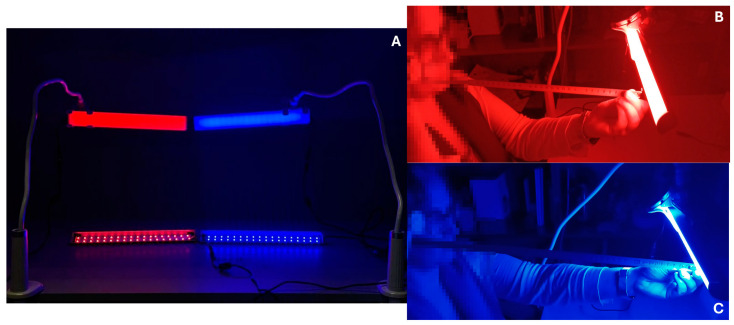
(**A**) Luminaires used in the experiments: at the top, the custom-built luminaires, and at the bottom, the luminaires without the diffuser, showing the LEDs. (**B**) Luminaire with red LEDs, calibrating the distance for the experiment. (**C**) Luminaire with blue LEDs, adjusted using a meter to ensure the correct distance.

Two custom-made luminaires, Figure 2, were then built with these LEDs to be used during the experiments, with the objective of providing a photopic illuminance level of 80 lux on the corneal plane of the participants. This was controlled using a calibrated luxmeter (model Delta-Ohm, HD2102.1 and LP471PHOT Probe, Senseca Italy Srl, Padua, Italy), and it was determined that the red LED was located 40 cm from the corneal plane and the blue LED was positioned 55 cm from the corneal plane, oriented at a 45° angle, from the same vertical plane.

### 2.2. Theoretical Background

Both the CIE and WELL documents address lighting’s impact on human health. The CIE Position Statement offers technical guidance on integrative lighting, while the WELL Building Standard v2 provides a framework for health-promoting environments. To quantify the biological effects of light, metrics such as mEDI from CIE S 026 and EML from WELL are used. Previous research shows that the equation EML = 1.104·mEDI links the two systems, derived from spectral data calculations [29].

**Figure 3:**
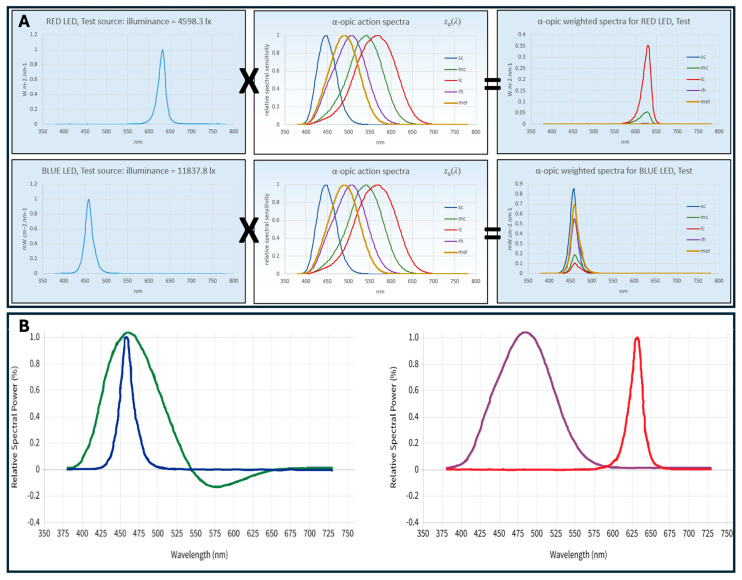
(**A**) Calculation of α-opic weighted spectra for red and blue LED test sources from CIE S 026 Toolbox (v1.49a—November 2020) [22]. The left figures show the spectral power distributions (SPDs) of the red LED top (illuminance = 4598.3 lx) and the blue LED bottom (illuminance = 11837.8 lx). The middle panels illustrate the α-opic action spectra for S-cones, M-cones, L-cones, rhodopic, and melanopic sensitivities. The right figures display the resulting α-opic weighted spectra for the red and blue LED test sources, obtained by multiplying the SPDs with the respective α-opic action spectra. (**B**) Calculation of the relative spectral contribution to the circadian response for cool (green) and warm (purple) lights, as well as blue and red LEDs, respectively, according to Rea et al. [26,27].

Meanwhile, the CS metric outlined in the “Design Guideline for Promoting Circadian Entrainment with Light for Day-Active People” (DG 24480) guideline recommends a target CS value of 0.3 for at least 2 h in the morning to promote circadian entrainment [26,27]. The required circadian light dose (CSd) of 0.43 can be achieved by varying the CL_A_ intensity or exposure duration, with a reciprocal relationship between the two within limits of 0.5–3 h [30]. The updated version of the CS calculation incorporates these parameters, enabling the calculation of CL_A_ based on any SPD and photopic illuminance, thus determining the exposure duration needed to meet the CSd = 0.43 target. These recent model revisions have refined the CL_A_ spectral sensitivity curve, preserving the correlation between CS and nocturnal melatonin suppression, updating the description for CL_A_ (CL_A_ 2.0) allowing to align with reference illuminants, yielding a CL_A_ of 813 at 1000 lux from CIE Illuminant A and adjusting equivalencies for diverse lighting conditions for improving accuracy [28], such as CIE Illuminant D65 (daylight at 6500 K). It was designed to maintain the relationship between CS and predicted nocturnal melatonin suppression (e.g., CS of 0.5 results in 50% nocturnal melatonin suppression following a reference light exposure duration of 1 h), and to achieve these values, CL_A_ 2.0 approximates photopic illuminance for general “white” light, with conversion factors of 1.23 for CIE Illuminant A and 0.66 for CIE daylight Illuminant D65 (Figure 3 and Table 2).

This revised circadian light model [26,27] optimized spectral sensitivity using photoreceptor fundamentals that differ slightly from CIE S 026 standards [22,23,31]. For melanopsin, the model uses the Wyszecki and Stiles [32] template, with a peak sensitivity at 485 nm and half-max sensitivity of 89 nm, rather than the CIE’s 490 nm peak and 84 nm half-max from Govardovskii et al. [33]. While this choice had minimal impact on broadband predictions, it significantly improved accuracy for narrowband sources, suggesting it better characterizes melanopsin’s in vivo action spectrum. This model indirectly tests the suitability of the CIE melanopic function for circadian sensitivity. The CL_A_ equation incorporates four unitless, normalized photoreceptor action spectra (M(λ), V(λ), V’(λ), and S-cone(λ)), each scaled to a maximum value of 1. These spectra represent in vivo photoreceptor sensitivities, accounting for preretinal filtering, such as lens absorption. The CIE photopic (V(λ)) and scotopic (V’(λ)) functions represent the L- and M-cone achromatic channel and the rod achromatic channel, respectively. Adjustments to the melanopsin (485 nm) response for lens transmission and the S-cone sensitivity for macular pigment removal required renormalizing each spectrum to a peak value of 1 [34,35]. This part is a different method from CIE, but it can also be computed from this standard when considering the transmittance of the crystalline lens depending on the age of the observer [36].

### 2.3. Sample Description

This study was conducted in accordance with the principles outlined in the Declaration of Helsinki and received approval from the Ethics Committee for Research of the Community of Aragón (CEICA), with registration code PI24/483. The participants provided informed consent prior to their inclusion in the study and were required to be healthy adults, aged 18 to 55, with no diagnosed ocular or systemic diseases and not undergoing pharmacological treatments that could affect melatonin secretion. Regarding clinical status, all participants self-reported good general health and were screened to exclude any history of neurological, psychiatric, sleep, or endocrine disorders. Besides, participants who were excluded from the experiment met one or more of the following exclusion criteria: presence of ophthalmological or systemic pathologies affecting vision; use of electronic devices one hour before the measurements, stimulating accommodation; consumption of coffee; smoking; and/or engaging in high-intensity sports activities.

### 2.4. Saliva Sample Collection

For saliva sample collection, participants did not eat, drink, chew gum, or brush their teeth for at least 30 min before sample collection. Samples were not taken if there were oral diseases, inflammations, or lesions to avoid blood contamination, as indicated by a reddish color in the saliva. Participants who had consumed multivitamins or supplements containing biotin within the previous 48 h were also excluded. During the study period, medications that affect melatonin secretion, such as benzodiazepines, fluvoxamine, caffeine, vitamin B12, and certain non-steroidal anti-inflammatory drugs, were avoided. Five minutes before collection, participants rinsed their mouths with cold water. A minimum of 0.5 mL of saliva was collected in an Eppendorf tube every hour. Samples were then stored at 2–8 °C for up to 5 days and delivered to the laboratory on ice, preferably dry ice, avoiding heat and direct sunlight. Once in the laboratory, samples were frozen at −20 °C for up to 2 weeks until analysis.

### 2.5. Experimental Protocol

The experiment was conducted on two separate days, each day with a different light source. Participants were exposed to the light (red or blue) in a random order to prevent any bias in the results. Regardless of which light was used first, once the setup was arranged with the appropriate distances for each illumination, participants remained under the light for 3 h without wearing glasses or contact lenses to avoid any wavelength interference. During this period, they were not allowed to use devices or screens of any kind, including mobile phones or tablets, nor watch television; only reading printed material was permitted. The experiment took place in March, from 9:00 p.m. to midnight, meaning that it was already dark at that time in the location of Zaragoza in Spain (41°38′31″ N, 0°53′60″ W; 243 m above sea level) to ensure participants were in the initial phase of melatonin secretion. After one hour under the designated light, at 10:00 p.m., participants collected a saliva sample in an Eppendorf tube, which was then stored in a refrigerator at the specified temperature. This process was repeated at the second (11:00 p.m.) and third hours (00:00 a.m.), resulting in a total of three saliva samples collected per light condition. For further information, four participants had saliva samples collected at 3:00 a.m. to evaluate the trend in melatonin secretion after sleeping, before being exposed to the lights.

### 2.6. Determination of Melatonin in Saliva Samples

Melatonin concentration in collected at predetermined time points saliva samples was determined using a direct high-sensitivity enzyme-linked immunosorbent assay (ELISA) kit for human saliva samples (Melatonin direct Saliva ELISA, Tecan, IBL International GmbH, Hamburg, Germany) according to the manufacturer’s protocol. For this purpose, prior to analysis, samples were thawed, mixed, and centrifuged at 2500× *g* for 10 min to remove particulate material. For each run, a melatonin standard curve (0–50 pg/mL) was used to calculate the melatonin concentration in the samples, expressed in pg/mL, and quality controls were included to validate the assay’s accuracy. Standards, controls, and samples were analyzed in duplicate to ensure reliability. Absorbance readings on the plates were performed using a Synergy™ Multi-Detection Microplate Reader (BioTek Instruments, Winooski, VT, USA) controlled with Gen5™ Data Analysis Software (version 3.04), measuring at 450 nm wavelength with 600 nm wavelength as reference.

### 2.7. Data Processing and Statistical Analysis

The absorbance data obtained for the standards in the ELISA assays were fitted to a four-parameter logistic curve (4PL) using the free online program MyCurveFit^®^ (MyAssays Ltd., Brighton, UK), from which the melatonin concentration of the samples was extrapolated. The data collected for the variables during the study were exported to Excel and processed using the Statistical Package for the Social Sciences (SPSS 24.0 Inc., Chicago, IL, USA) for analysis. Descriptive statistics, including the mean and standard error (SE), were calculated for the numerical variables. The Kolmogorov-Smirnov test indicated that the variables did not follow a normal distribution, prompting the use of non-parametric tests for related samples. The Wilcoxon signed-rank test was employed to assess differences in melatonin concentration between the two lighting conditions, with statistical significance set at a *p*-value < 0.05.

## 3. Results

The sample consisted of 12 participants, among whom seven (58.33%) were women and five (41.67%) were men, ranging from 19 to 55 years with a mean age of 30.08 ± 12.91 years, all of whom met the inclusion criteria.

### 3.1. Melatonin Concentration Across All Participants

**Figure 4:**
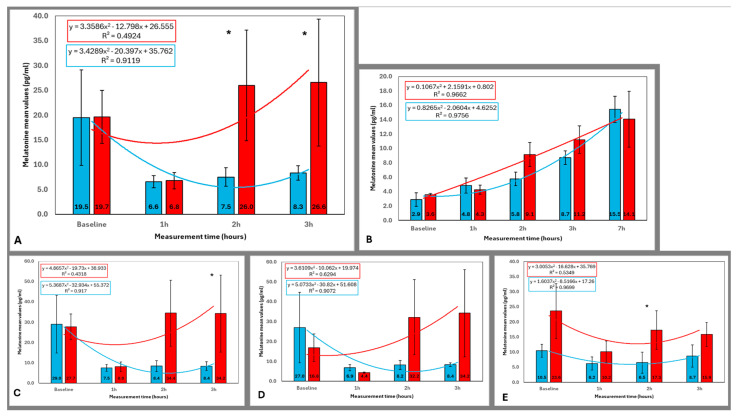
Mean (± standard deviation) melatonin concentration (pg/mL) across the four time points (baseline, 1 h, 2 h, and 3 h) under blue and red light conditions for different participant groups: (**A**) all participants (n = 12), (**B**) older female participants (n = 4, ages 32–55), including a 7-h measurement at 3 a.m., (**C**) young participants (n = 8), (**D**) all female participants (n = 7), and (**E**) all male participants (n = 5). The *X*-axis represents the time points of saliva sample collection, and the *Y*-axis shows the mean melatonin concentration for each lighting condition. Trend lines with equations illustrate melatonin secretion over time for both lighting conditions. Significant differences (*p* < 0.05) between blue and red light conditions at the same specific time points are marked with an asterisk (*). Statistical analysis was performed using the Wilcoxon signed-rank test for related samples.

The analysis included all 12 participants, comparing mean salivary melatonin concentrations under blue and red light conditions at baseline and after 1, 2, and 3 h of exposure (Figure 4A). At baseline, melatonin levels were similar on the day of exposure to blue light (19.5 pg/mL) and red light (19.7 pg/mL), with no significant difference (*p* = 1.00). After 1 h of exposure, both lighting conditions resulted in a notable decrease in melatonin concentration, with levels of 6.6 pg/mL under blue light and 6.8 pg/mL under red light, without any significant difference between them (*p* = 0.754). Significant differences between the two lighting conditions emerged after 2 h of exposure. Melatonin concentration under blue light was 7.5 pg/mL, whereas it increased to 26.0 pg/mL under red light, yielding a statistically significant difference (*p* = 0.019). This difference persisted after 3 h of exposure, with melatonin levels of 8.3 pg/mL under blue light and 16.6 pg/mL under red light (*p* = 0.013). These results indicate that blue light significantly suppresses melatonin secretion after 2 h of exposure, highlighting its impact on the circadian rhythm.

### 3.2. Melatonin Concentration in Older and Younger Participants

The results for the 4 older female participants, aged between 32 and 55 years, with a mean age of 46 ± 9.83 years, were calculated separately (Figure 4B). In the baseline measurement, the melatonin concentrations were 2.9 pg/mL under blue light and 3.6 pg/mL under red light (*p* = 0.465). After 1 h of exposure, the concentrations were 4.8 pg/mL with blue light and 4.3 pg/mL with red light (*p* = 0.465). After 2 h, the melatonin levels were 5.8 pg/mL under blue light and 9.1 pg/mL under red light (*p* = 0.068), approaching statistical significance. At 3 h, the concentrations were 8.7 pg/mL with blue light and 11.2 pg/mL with red light (*p* = 0.285). Finally, at 7 h (coinciding with 3 a.m., when melatonin concentration typically peaks), the melatonin levels were 15.5 pg/mL with blue light and 14.1 pg/mL with red light (*p* = 0.715). No statistically significant differences were found between the lighting conditions at any time point, although at 2 h of exposure, the difference between blue and red light approached significance (*p* = 0.068), with higher melatonin concentrations observed under red light. By 7 h, the concentrations were similar between the two light conditions.

The group of young participants (Figure 4C) included 8 individuals (5 men and 3 women) with a mean age of 22.13 ± 1.81 years. Baseline melatonin concentrations were 29.0 pg/mL for the blue light condition and 27.7 pg/mL for the red light condition (*p* = 1). After 1 h of exposure, melatonin levels decreased to 7.5 pg/mL under blue light and 8.0 pg/mL under red light (*p* = 0.889). At 2 h, concentrations were 8.4 pg/mL for blue light and 34.4 pg/mL for red light, with the difference approaching statistical significance (*p* = 0.069). By the 3-h mark, melatonin levels were 8.4 pg/mL for blue light and 34.2 pg/mL for red light, showing a statistically significant difference in melatonin concentrations between the two lighting conditions (*p* = 0.028). These results indicate a clear suppression of melatonin secretion under blue light exposure at 2 and 3 h in young participants, as illustrated in Figure 4C.

### 3.3. Melatonin Concentration in Female and Male Participants

The sample of female participants was made up of 7 women who had a mean age of 36 ± 14.4 years, ranging from 19 to 55 years. Baseline melatonin concentrations in saliva were 27.0 pg/mL under blue light and 16.8 pg/mL under red light, with no significant difference between the two (*p* = 0.463). After 1 h of exposure, melatonin concentrations were 6.9 pg/mL with blue light and 4.4 pg/mL with red light (*p* = 0.128). After 2 h, the concentrations were 8.2 pg/mL with blue light and 32.2 pg/mL with red light (*p* = 0.128). At 3 h, concentrations were 8.4 pg/mL with blue light and 34.2 pg/mL with red light (*p* = 0.116). No significant differences in melatonin concentrations between the blue and red light conditions were observed. However, as shown in Figure 4D, after 2 h of exposure to blue light, melatonin concentration in saliva was notably lower, a trend that persisted after 3 h of exposure.

The male group consisted of 5 participants (Figure 4E) with a mean age of 21.8 ± 0.45 years, ranging between 21 and 22 years. At baseline, the melatonin concentration in saliva was 10.5 pg/mL under blue light and 23.6 pg/mL under red light, with no significant difference (*p* = 0.225). After 1 h of exposure, the concentrations were 6.2 pg/mL with blue light and 10.2 pg/mL with red light (*p* = 0.225). After 2 h, concentrations were 6.5 pg/mL with blue light and 17.3 pg/mL with red light, showing a statistically significant difference (*p* = 0.043), with higher melatonin levels under red light. At 3 h, the concentrations were 8.7 pg/mL with blue light and 15.9 pg/mL with red light, nearing statistical significance (*p* = 0.068). As shown in Figure 4E, melatonin concentrations increased significantly under red light exposure at the 2-h time point and approached significance at the 3-h time point, while blue light exposure showed consistently lower melatonin concentrations.

### 3.4. Comparison of Melatonin Levels Across Time Points for Each Lighting Condition

In Table 3, the *p*-values obtained from the Wilcoxon signed-rank test for paired samples are presented. These values compare the melatonin concentrations after 1 h of exposure to each lighting condition (blue and red) with the melatonin levels recorded at baseline, as well as the concentrations after 2 and 3 h of each exposure.

The choice of 1 h as the reference point for comparison was made because, across all participant groups, this is the time point where the most pronounced decrease in salivary melatonin concentration was observed, regardless of the lighting condition.

After 1 h of exposure to blue light, a substantial decrease in salivary melatonin concentration was observed when considering all participants together (*p* = 0.182), the group of young participants (*p* = 0.063), the group of women (*p* = 0.917), and the group of men (*p* = 0.225) compared to baseline levels. Melatonin levels increased slightly after 2 and 3 h of exposure in all cases, as shown in Figure 4A,C–E, respectively, without reaching statistical significance in any group (Table 3).

In contrast, after 1 h of exposure to red light, a significant decrease in salivary melatonin concentration compared to baseline was observed when considering all participants together (*p* = 0.041) and the group of young participants (*p* = 0.025). Although decreases were also noted in the group of women (*p* = 0.237) and the group of men (*p* = 0.138), these did not reach statistical significance. Furthermore, melatonin levels significantly increased in all participants and nearly all subgroups after 2 and 3 h of exposure (Table 3), as illustrated in Figure 4A,C–E.

## 4. Discussion

This study examined the effects of blue and red LED light on salivary melatonin concentrations, focusing on how their spectral characteristics influence circadian regulation. The findings highlight the significant role of the light spectrum in modulating melatonin secretion.

Blue light (peak 464 nm, overlapping with the melanopsin action spectrum) showed greater circadian stimulation and stronger, time-dependent melatonin suppression, particularly after 2 h. In contrast, red light (peak 631 nm, minimal overlap) preserved higher melatonin levels, indicating less circadian disruption. These differences may be influenced by prior light exposure, which modulates melatonin responses, with the greatest suppression occurring early and diminishing over time [16,18].

This study highlights age-specific differences in the effects of blue and red light on melatonin suppression. In older participants, no significant differences were observed between light conditions, though red light preserved slightly higher melatonin levels after 2 h. In younger participants, blue light significantly suppressed melatonin compared to red light, particularly after 2 and 3 h. Age-related declines in melatonin production have been attributed to factors such as reduced pupil size and lens yellowing, which limit retinal light exposure [16,19]. These changes lead to less light reaching the retina, which could theoretically impact circadian regulation, especially since retinal ganglion cells are more sensitive to blue light. However, in the context of our study, we consider the influence of these age-related ocular changes to be minimal. This is due to the substantial difference in the circadian effectiveness of light in the blue versus red spectrum selected to perform the experiments. Consequently, these pupillary changes, while important in a broader physiological context, seem to have limited influence on the circadian effects observed in our experiment, as reported by other authors [17].

The analysis by gender showed that in women, melatonin levels tended to be lower under blue light after 2 and 3 h, suggesting stronger circadian activation. However, the differences between light conditions were not significant, aligning with previous inconclusive findings [19]. For men, melatonin levels were significantly higher under red light after 2 h and approached significance at 3 h, while blue light consistently suppressed melatonin more, highlighting blue light’s stronger impact and red light’s circadian-friendly effects in both groups. These subgroup analyses were exploratory and based on a small sample, so the findings should be interpreted with caution. Larger and more balanced studies are needed to confirm these trends.

Globally, our results confirm red light’s circadian-friendly properties, particularly for females and younger participants, while blue light consistently suppressed melatonin across groups. This variability may stem from factors such as age-related ocular changes, pupil size differences, health status, medications, hormonal levels, and genetic variations affecting melanopsin pathways, as well as long-term light exposure patterns, such as seasonal changes [18,19]. Achieving 250 mEDI outdoors during the day is feasible, but evening exposure should be limited to 10 mEDI, with near-darkness at night (very low mEDI) being crucial for maintaining a healthy circadian rhythm, improving sleep quality, and enhancing overall well-being [14,23]. While this may conflict with visibility needs, it is recommended in integrative lighting design. Existing guidelines, based on healthy young adults, need to be extended to other populations, with future research focusing on personal light dosimetry to better understand exposure patterns. Current lighting standards, based on averages, often overlook individual variability [15]; as sensitivity to light can differ by up to 50–60 times [37], with some people suppressing melatonin at minimal light levels while others require much higher intensities, the need for flexible controls and tailored recommendations could be necessary for individuals active at night, taking into consideration their distinct activities and light exposure outside of work.

NIF functions vary in response speed, with rapid pupil reactions and slower EEG Gamma responses, highlighting individual variability in how light affects cognitive alertness and brain activity [38]. Recent findings show that non-visual response systems, including those involving the brain, cardiovascular system, and thermoregulation, activate within 1 to 5 min of light exposure, suggesting a faster response than previously thought [38]. Blue light enhances NIF effects, such as brain activation, while blocking green light may amplify polychromatic white light’s impact [39,40]. Psychological and behavioral responses to light also differ widely due to individual exposure and subjective alertness. Understanding these differences is crucial for tailoring light therapies to improve cognitive function, mood, and quality of life, especially in older populations. These variations arise because light impacts mood and behavior through ipRGCs, which mediate its effects on behavioral states [41]. However, the relationship between light exposure and daytime alertness varies significantly, with most dose-response analyses showing non-significant correlations [42].

Moreover, melanopic illuminance has become a key metric for predicting circadian effects, offering a valuable tool for assessing light’s impact on human physiology [24]. Our findings show that blue light suppresses melatonin within 1 h of exposure, maintaining this suppression with minimal recovery over the next hours. In contrast, red light also suppresses melatonin initially but allows a significant rebound after 2 and 3 h, restoring secretion to higher levels. This suggests red light may be beneficial for preserving circadian regulation. Additionally, light color affects both physiological and psychological responses; blue light is associated with relaxation, while red light evokes alertness or even danger, which might initially inhibit melatonin. However, as the brain adapts, this effect diminishes, leading to melatonin rebound. It is also important to note that our results may be influenced by the S-cone response, which overlaps with the melatonin absorption spectrum, and their mutual influence is still under discussion [10,43]. These results also emphasize the need for refined methodologies to assess circadian light hygiene, beyond static or solely melanopic-based metrics. While recent tools such as CircaLight offer reliable spectral-spatial simulations of circadian metrics [44], and conceptual frameworks [45] emphasize the importance of spatial, spectral, and temporal components in light assessment over 24 h, our findings contribute complementary biological evidence by characterizing melatonin suppression and rebound in response to spectrally distinct light over time. This time-resolved physiological approach may support future developments in circadian light hygiene assessment by integrating not only spectral and spatial, but also temporal responsiveness of human physiology to light.

Existing lighting guidelines are primarily based on healthy young adults, indicating a need for further research to adapt these recommendations for diverse populations, particularly older adults or those with disrupted circadian rhythms. Future studies should focus on personal light dosimetry to better understand individual light exposure patterns. Additionally, current lighting standards often overlook individual variability, highlighting the need for adaptable lighting controls and tailored recommendations, especially for individuals active at night. While these findings offer valuable insights, more research is needed to fully understand the mechanisms involved and optimize lighting strategies.

## 5. Conclusions

In conclusion, our findings demonstrate that blue light causes a stronger and more sustained melatonin suppression than red light, highlighting its significant impact on the circadian system. In contrast, red light, while initially lowering melatonin, allowed for notable recovery, suggesting it is less disruptive to circadian rhythms. These results underline the importance of the light spectrum in circadian regulation and suggest red light as a potential option to reduce circadian disruption. This study represents a preliminary step toward understanding these effects, and future research should include a larger and more diverse sample to validate these findings, particularly with respect to age and gender differences.

## Figures and Tables

**Table 1:** Properties of two LED light conditions (Blue and Red) at an illuminance level of 80 lx, including Irradiance, and chromaticity coordinates (x, y).

	Blue	Red
Irradiance (W·m^−2^)	1.628	0.407
Chromaticity Coordinates (x, y)	(0.1438, 0.0389)	(0.6898, 0.3068)

**Table 2:** Spectral and photometric characteristics under blue and red light conditions at an illuminance level of 80 lx on the corneal plane, including α-opic efficacy ratios, melanopic equivalent daylight (D65) illuminance (mEDI), equivalent melanopic lux (EML), melanopic ratio (M/P), circadian stimulus (CS), and circadian light (CL_A_ 2.0).

Illuminance 80 lx			Blue	Red
CIES026 [22,23,31]	Irradiance (W·m^−2^)	S-cone	1.256	6.71 × 10^−4^
		M-cone	0.339	3.23 × 10^−2^
		L-cone	0.196	1.59 × 10^−1^
		Rhodopic	0.913	3.76 × 10^−3^
		Melanopic	1.123	1.42 × 10^−3^
	α-opic DER for (α-opic daylight (D65) efficacy ratio is 1.000)	S-cone-opic	19.2131	0.0103
		M-cone-opic	2.9101	0.2775
		L-cone-opic	1.5070	1.2199
		Rhodopic	7.8763	0.0324
		Melanopic	10.5823	0.0134
	$E_{v,\alpha }^{D65}$ Melanopic Equivalent Daylight (D65) Illuminance [lux]; mEDI		847	1
WELL [25]	Equivalent Melanopic Lux [m-lux]; EML		934	1
	Melanopic Ratio, R (M/P)		11.68	0.01
Rea et al. [26,27]	CS		0.588	0.003
	CL_A_ 2.0		1600	2

**Table 3:** *p*-values obtained using the Wilcoxon signed-rank test for paired samples, comparing melatonin concentrations at 1 h of exposure to blue and red light with baseline, 2 h, and 3 h melatonin levels for each lighting condition (blue and red). The analysis was conducted separately for each participant group. A *p*-value < 0.05 was considered statistically significant, and significant results are highlighted in bold with an asterisk (*).

Group	Blue Light 1 h vs.			Red Light 1 h vs.		
	Baseline	2 h	3 h	Baseline	2 h	3 h
All (n = 12) Figure 4A	0.182	0.583	0.386	0.041 *	0.028 *	0.012 *
Younger (n = 8) Figure 4B	0.063	1.000	0.735	0.025 *	0.093	0.093
Older (n = 4) Figure 4C	0.068	0.273	0.109	0.465	0.068	0.068
Female (n = 7) Figure 4D	0.917	0.398	0.249	0.237	0.018 *	0.028 *
Male (n = 5) Figure 4E	0.225	0.686	0.715	0.138	0.500	0.225

## Data Availability

The data presented in this study are available within the article.

## Abbreviations

The following abbreviations are used in this manuscript:

SCN	Suprachiasmatic nucleus
RHT	Retinohypothalamic tract
ipRGCs	Intrinsically photosensitive retinal ganglion cells
NIF	Non-image-forming
PLR	Pupillary light reflex
CIE	Commission International de l’Eclairage
mEDI	Melanopic equivalent daylight (D65) illuminance
EML	Equivalent melanopic lux
CL_A_	Circadian Light
CS	Circadian stimulus
SPD	Spectral power distribution
FWHM	Full width at half maximum
CSd	Circadian light dose
SE	Standard error

## References

[1] Wahl S., Engelhardt M., Schaupp P., Lappe C., Ivanov I.V. (2019). The Inner Clock—Blue Light Sets the Human Rhythm. J. Biophotonics.

[2] Pundir M., Papagerakis S., De Rosa M.C., Chronis N., Kurabayashi K., Abdulmawjood S., Prince M.E.P., Lobanova L., Chen X., Papagerakis P. (2022). Emerging Biotechnologies for Evaluating Disruption of Stress, Sleep, and Circadian Rhythm Mechanism Using Aptamer-Based Detection of Salivary Biomarkers. Biotechnol. Adv..

[3] Figueiro M.G., Nagare R., Price L.L.A. (2018). Non-Visual Effects of Light: How to Use Light to Promote Circadian Entrainment and Elicit Alertness. Light. Res. Technol..

[4] Berson D.M., Dunn F.A., Takao M. (2002). Phototransduction by Retinal Ganglion Cells That Set the Circadian Clock. Science (1979).

[5] Foster R.G. (2021). Fundamentals of Circadian Entrainment by Light. Light. Res. Technol..

[6] Provencio I., Rodriguez I.R., Jiang G., Hayes W.P., Moreira E.F., Rollag M.D. (2000). A Novel Human Opsin in the Inner Retina. J. Neurosci..

[7] Thapan K., Arendt J., Skene D.J. (2001). An Action Spectrum for Melatonin Suppression: Evidence for a Novel Non-Rod, Non-Cone Photoreceptor System in Humans. J. Physiol..

[8] Brainard G.C., Hanifin J.P., Greeson J.M., Byrne B., Glickman G., Gerner E., Rollag M.D. (2001). Action Spectrum for Melatonin Regulation in Humans: Evidence for a Novel Circadian Photoreceptor. J. Neurosci..

[9] Lucas R.J., Peirson S.N., Berson D.M., Brown T.M., Cooper H.M., Czeisler C.A., Figueiro M.G., Gamlin P.D., Lockley S.W., O’Hagan J.B. (2014). Measuring and Using Light in the Melanopsin Age. Trends Neurosci..

[10] Brown T.M., Thapan K., Arendt J., Revell V.L., Skene D.J. (2021). S-Cone Contribution to the Acute Melatonin Suppression Response in Humans. J. Pineal. Res..

[11] Novotny P., Plischke H. (2017). Pupillary Light Reflex and Circadian Synchronization in the Elderly. Psych. J..

[12] Mure L.S. (2021). Intrinsically Photosensitive Retinal Ganglion Cells of the Human Retina. Front. Neurol..

[13] Najjar R.P., Prayag A.S., Gronfier C. (2024). Melatonin Suppression by Light Involves Different Retinal Photoreceptors in Young and Older Adults. J. Pineal. Res..

[14] Brown T.M., Brainard G.C., Cajochen C., Czeisler C.A., Hanifin J.P., Lockley S.W., Lucas R.J., Münch M., OHagan J.B., Peirson S.N. (2022). Recommendations for Daytime, Evening, and Nighttime Indoor Light Exposure to Best Support Physiology, Sleep, and Wakefulness in Healthy Adults. PLoS Biol..

[15] Chellappa S.L. (2021). Individual Differences in Light Sensitivity Affect Sleep and Circadian Rhythms. Sleep.

[16] Nagare R., Plitnick B., Figueiro M.G. (2019). Effect of Exposure Duration and Light Spectra on Nighttime Melatonin Suppression in Adolescents and Adults. Light. Res. Technol..

[17] Eto T., Higuchi S. (2023). Review on Age-Related Differences in Non-Visual Effects of Light: Melatonin Suppression, Circadian Phase Shift and Pupillary Light Reflex in Children to Older Adults. J. Physiol. Anthropol..

[18] Lee S.-I., Kinoshita S., Noguchi A., Eto T., Ohashi M., Nishimura Y., Maeda K., Motomura Y., Awata Y., Higuchi S. (2020). Melatonin Suppression during a Simulated Night Shift in Medium Intensity Light Is Increased by 10-Minute Breaks in Dim Light and Decreased by 10-Minute Breaks in Bright Light. Chronobiol. Int..

[19] Swope C.B., Rong S., Campanella C., Vaicekonyte R., Phillips A.J.K., Cain S.W., McGlashan E.M. (2023). Factors Associated with Variability in the Melatonin Suppression Response to Light: A Narrative Review. Chronobiol. Int..

[20] Ju J., He L., Li Z., Wu Z., He Z., Liang R. (2012). Biological Effects of Color Lighting for Different Ages. Appl. Mech. Mater..

[21] Reutrakul S., Crowley S.J., Park J.C., Chau F.Y., Priyadarshini M., Hanlon E.C., Danielson K.K., Gerber B.S., Baynard T., Yeh J.J. (2020). Relationship between Intrinsically Photosensitive Ganglion Cell Function and Circadian Regulation in Diabetic Retinopathy. Sci. Rep..

[22] (2018). CIE System for Metrology of Optical Radiation for IpRGC-Influenced Responses to Light.

[23] (2024). Position Statement CIE Position Statement on Integrative Lighting Recommending Proper Light at the Proper Time.

[24] Brown T.M. (2020). Melanopic Illuminance Defines the Magnitude of Human Circadian Light Responses under a Wide Range of Conditions. J. Pineal. Res..

[25] IWBI International Well Building Institute (2021). WELL Building Standard v2 Pilot, Q3 2021 Version. Section L03: Circadian Lighting Design.

[26] Rea M.S., Nagare R., Figueiro M.G. (2021). Modeling Circadian Phototransduction: Quantitative Predictions of Psychophysical Data. Front. Neurosci..

[27] Rea M.S., Nagare R., Figueiro M.G. (2021). Modeling Circadian Phototransduction: Retinal Neurophysiology and Neuroanatomy. Front. Neurosci..

[28] Rea M.S., Figueiro M.G., Bullough J.D., Bierman A. (2005). A Model of Phototransduction by the Human Circadian System. Brain Res. Rev..

[29] Sánchez-cano A., Aporta J. (2020). Optimization of Lighting Projects Including Photopic and Circadian Criteria: A Simplified Action Protocol. Appl. Sci..

[30] Schlangen L.J.M., Belgers S., Cuijpers R.H., Heynderickx I.E.J. (2022). Invited Comment on “The Law of Reciprocity Holds (More or Less) for Circadian-Effective Lighting”, by M Rea, Accepted for Publication in LRT 2022. Light. Res. Technol..

[31] (2019). CIE Position Statement on Non-Visual Effects of Light Recommending Proper Light at the Proper Time.

[32] Wyszecki G., Stiles W.S. (2000). Color Science: Concepts and Methods, Quantitative Data and Formulae.

[33] Govardovskii V.I., Fyhrquist N., Reuter T.O.M., Kuzmin D.G., Donner K. (2000). In Search of the Visual Pigment Template. Vis. Neurosci..

[34] Smith V.C., Pokorny J. (1975). Spectral Sensitivity of the Foveal Cone Photopigments between 400 and 500 Nm. Vis. Res..

[35] Commission International de l’Eclairage (CIE) (1978). Light as a True Visual Quantity: Principles of Measurement.

[36] Sanchez-Cano A., Orduna-Hospital E., Fernández-Espinosa G., Aporta J. (2023). Method to Calculate Melanopic Light Reaching the Retina Depending on the Optical Density of an Aging Crystalline Lens. Appl. Sci..

[37] Phillips A.J.K., Vidafar P., Burns A.C., McGlashan E.M., Anderson C., Rajaratnam S.M.W., Lockley S.W., Cain S.W. (2019). High Sensitivity and Interindividual Variability in the Response of the Human Circadian System to Evening Light. Proc. Natl. Acad. Sci. USA.

[38] Prayag A.S., Jost S., Avouac P., Dumortier D., Gronfier C. (2019). Dynamics of Non-Visual Responses in Humans: As Fast as Lightning?. Front. Neurosci..

[39] Morioka H., Ozawa H., Kato T. (2023). Physiological Study of Visual and Non-Visual Effects of Light Exposure. Appl. Sci..

[40] Lee S., Kakitsuba N., Katsuura T. (2018). Do Green-Blocking Glasses Enhance the Nonvisual Effects of White Polychromatic Light?. J. Physiol. Anthropol..

[41] Milosavljevic N. (2019). How Does Light Regulate Mood and Behavioral State?. Clocks Sleep.

[42] van Duijnhoven J., Aarts M.P.J., van den Heuvel E.R., Kort H.S.M. (2021). Exploring the Relationship between Light and Subjective Alertness Using Personal Lighting Conditions. J. Phys. Conf. Ser..

[43] Fazlali F., Lazar R., Yahya F., Epple C., Spitschan M., Stefani O., Cajochen C. (2024). Lack of Evidence for the Contribution of Cone Photoreceptors to Human Melatonin Suppression and Alerting Response to Light at Night. bioRxiv.

[44] Aguilar-Carrasco M.T., Acosta I., Domínguez-Amarillo S. (2023). CircaLight, a New Circadian Light Assessment Tool for Grasshopper Environment: Development and Reliability Testing. J. Build. Eng..

[45] Gubin D.G., Borisenkov M.F., Kolomeichuk S.N., Markov A.A., Weinert D., Cornelissen G., Stefani O. (2024). Evaluating Circadian Light Hygiene: Methodology and Health Implications. Russ. Open Med. J..

